# Myxoid liposarcoma: a rare soft-tissue tumor with a misleading benign appearance

**DOI:** 10.1186/1477-7819-7-42

**Published:** 2009-04-22

**Authors:** Francois Loubignac, Christophe Bourtoul, Francoise Chapel

**Affiliations:** 1 Orthopaedic and traumatology Surgery "A", Font-Pré Hospital, Toulon, France; 2 Visceralous Surgery, Font-Pré Hospital, Toulon, France; 3 Anatomopathology Department, Font-Pré Hospital, Toulon, France

## Abstract

**Background:**

Lipoma is by far the most common of all benign soft-tissue tumors which far outnumber malignant tumors. Soft-tissue sarcomas are malignant tumors and are usually named for the type of tissue in which they begin. Liposarcoma (LPS), which arises in the fatty tissue, is rather an uncommon soft-tissue tumor. Multiple histologic subtypes of liposarcoma are recognized, including myxoid liposarcoma, and correspond to tumors of very different prognosis. In two-third of the cases, this tumor occurs in the muscle while often demonstrating a misleading benign appearance as observed in the majority of soft-tissue sarcomas.

**Case presentation:**

We report the case of a 50-year-old man operated on for a fat tumor of the thigh initially diagnosed as lipoma but revealing to be a myxoid liposarcoma after histopathological examination. The initial incomplete tumor excision required the need for a re-excision with adjuvant chemotherapy and complementary radiotherapy.

**Conclusion:**

When any suspicious soft-tissue tumor is diagnosed, the combined information gathered from accurate preoperative radiographic planning and X-rays or surgical biopsy is of tremendous value for establishing the most appropriate therapeutic program, highly adapted to the histopathological findings.

## Background

Lipomas account for 50% of all benign soft-tissue tumors. Malignant tumors or sarcomas comprise approximately 1% of all soft-tissue tumors. They are also rare among the malignant tumors that occur in adult, reporting a prevalence lower than 1% and an incidence of 30 cases per million population [1,2]. Liposarcoma itself (LPS) comprises about 15% of all soft-tissue sarcomas in the adult and its prognosis is highly related to the location, and more particularly to the histologic pattern of the tumor [3-5]. This fat tumor, of ubiquitous localization, commonly appears as a slowly enlarging mass with a misleadingly benign appearance [6]. However, any soft-tissue tumor requires the need for a thorough preoperative X-Ray investigation [1,7] and a biopsy should be performed if of more than five centimeters diameter [2].

## Case presentation

Mr A. Oma, a patient aged 50 years, with a 5-year history of a small tumorous lesion, and regularly followed after a polytraumatism, presents on 23 December 2003, with the complaint of a gradually growing mass in his thigh for three months. This small tumor involving the antero-lateral aspect of the thigh within the upper one-third, has a soft consistency, no adherences to the surrounding structures and is well circumscribed. Neither functional nor general symptoms are associated and routine blood tests are normal. Such features suspects a common adipose tumor of lipoma type and its excision is scheduled on 27 January 2004. This simple and rapid surgical procedure includs the excision of an intramuscular, subaponeurosis tumor, very well defined within the vast external of the crural quadriceps. The tumor of approximately 8 cm is sent to the laboratory of pathological anatomy. The excised specimen is a 7.5-cm, well defined, flexible, ovoid tumor of gelatinous aspect and brownish appearance, with some more fleshy zones, without necrotic or hemorrhagic changes.

**Figure 1:**
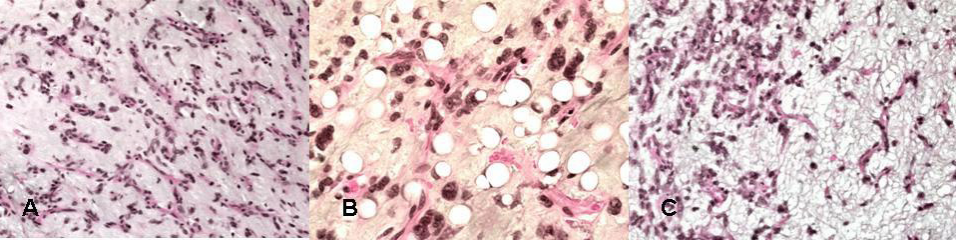
**Photomicrograph 1A, 1B, 1C: histopathologic aspects of myxoid and round cell liposarcoma**.

Histologic examination reveals a myxoid-type tumor, consisting of an amorphous mucoid material, slightly colored with small dark oval cells and no evidence of atypia or mitotic figures (fig. 1A). An extensive capillary network combined with the presence of some characteristic lipoblastic cells confirms the diagnosis of liposarcoma (fig. 1B). The tumor exhibits areas of variable cellular density; areas of dense proliferation of slightly atypical round cells and rare mitotic figures (5 mitoses/10 fields × 40) are detected (fig. 1C). The proportion of round cells is hardly definable, and approximates 20%. The tumor is partially encapsulated and situated less than one millimeter from the margins of excision and is associated with some satellite micro-nodules. The tumor is pathologically diagnosed as myxoid/round-cell liposarcoma, of histoprognostic grade 2 according to the National Union of Cancer Centers grading system (NUCCGS) (differentiation: grade 3, mitotic index: grade 1 and necrosis: grade 0), and involves the margins of resection [8,9]. A loco-regional assessment of tumoral extension by MRI Scan of the thigh, and a general assessment by thoracic and abdominal-CT-TAP-Scan are performed and reveal negative. The oncology committee of the Paoli Calmette Institute of Marseille thus recommends a re-excision procedure which is performed on March 11th, 2004. All tissue potentially exposed to viable tumor cells at the initial procedure have to be removed during revision surgery including the surgical scar, the path of the Jost-Redon drain, then, as a whole, the fascia and deeper muscular structures on the entire height of the initial tumor bed while performing a two centimeter-wider margin of excision. The histopathology examination does not reveal any tumor residues at the site of tumor bed excision.

Postoperative management is simple: from March to July 2004, the patient is treated with adjuvant chemotherapy consisting of six cycles of MAID-type [Doxorubicine (20 mg/m^2^), Ifosfamide (2500 mg/m^2^), Dacarbazine (300 mg/m^2^) and Mesna (6000 mg)] combined with radiotherapy delivering 50 Gy on the left thigh followed by 10 Gy focused on the operating site.

In November 2004, the clinical and paraclinic post-therapeutic follow-up (MRI scan and CT-Scan) reveal normal with minor orthopedic sequelas; a schedule of radio-clinical follow-up is established (MRI Scan and TAP-CT-Scan); the patient is in clinical remission at five years.

## Discussion

Despite the misleading benign appearance of this malignant tumor, complete preoperative X-Ray investigations and biopsy should have been performed since any deep tumor of soft tissues and/or which size exceeds five centimeters is considered as being suspect and requires biopsy prior to any excision procedure. Biopsy plays a crucial role in accurate histopathological diagnosis, and proper staging would enable the oncology committee to implement the most appropriate therapeutic protocol [2]. The first biopsy must be guided by ultrasound or CT-Scan; in case of failure, a surgical biopsy will be indicated and performed through a suitable surgical approach to avoid compromising the subsequent conservative management. In the present case, the evidence of positive excision margins increases the risk of tumor recurrence (around 60%) in the absence of re-excision [2]. This resumption must be scheduled from the results of a MRI Scan which looks for potential tumor anatomical residue and should be performed once proper healing of the initial surgery has been achieved. The re-excision procedure takes place at the initial site of resection and is performed with a wider margin of at least two centimeters of healthy tissue. All tissue potentially exposed to viable tumor cells at the initial procedure should be removed at that time, including the surgical scar and the orifices of drainage. A complementary radiation therapy should be implemented on the operating site, with a minimal safety margin of five centimeters, combined with adjuvant chemotherapy [10,11]. Lipomas and liposarcomas are of adipose origin according to the histogenetic classification of the World Health Organization [8,9,12]. Soft-tissue sarcomas are malignant tumors that originate in the soft tissues of the body which include muscle, fat, fibrous tissue, blood vessels or peripheral nerves.

The genesis of soft-tissue sarcomas has not yet been clearly defined but several contributing factors that increase the likelihood of developing these tumors have been identified: external radiation therapy and genetic factors are the most well-established risk factors for soft tissue sarcomas. Approximately 1% of patients treated with radiation therapy for a malignant tumor, might develop, in previously irradiated tissues, an osseous or soft tissue "radiation-induced sarcoma", which may appear from three to ten years later [10]. Some genetic diseases (neuromatosis, retinoblastoma, L-Fraumeni syndrome) may lead to the development of soft-tissue sarcomas. Lipoma is a very common tumor, generally, of a small size (less than 5 cm) and of superficial aspect whereas liposarcoma is a much rarer tumor of large (more than 5 cm) deep-seated connective tissue spaces, most commonly originating (three out of four times) under the superficial fascia [1]. Three main histological sub-types of LPS are recognized, which differ in their morphological aspect and evolution: well-differentiated LPS (the most frequent), Myxoid LPS and\or with round cells (40% of the LPS), and the anaplastic LPS, a rarer category of bad prognosis [3,8]. Myxoid LPS and round-cell LPS represent the same entity since they share a key genetic defect (t12; 16), (q13; p11). This genetic abnormality results in fusion of the transcription factor gene CHOP with FUS, and might be discovered through specific techniques (RT-PCR or FISH) while playing a critical role in the differential diagnosis [12,13]. Actually, the myxoid LPS may contain a variable number of round cells which determines the degree of differentiation and affects the prognosis [4]. There is still no consensus on the percentage of round cells which would help in the grading of such tumors; nevertheless, any myxoid LPS containing more than 10% of round cells should conduct to a cautious prognosis due to the risk of metastases occurrence [4,8]. The myxoid liposarcoma occurs predominantly at the level of the muscular chamber of the limbs and more specifically in the thigh in more than 2/3 of the cases; it rarely occurs in the retroperitoneum or the subcutaneous tissue.

**Figure 2:**
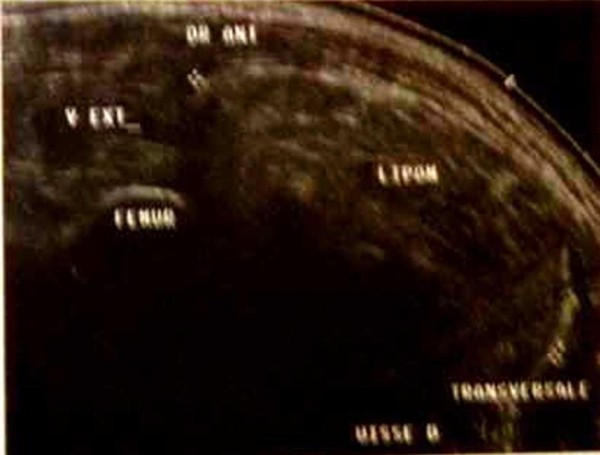
**ultrasound aspect of a deep lipoma of the thigh**.

**Figure 3:**
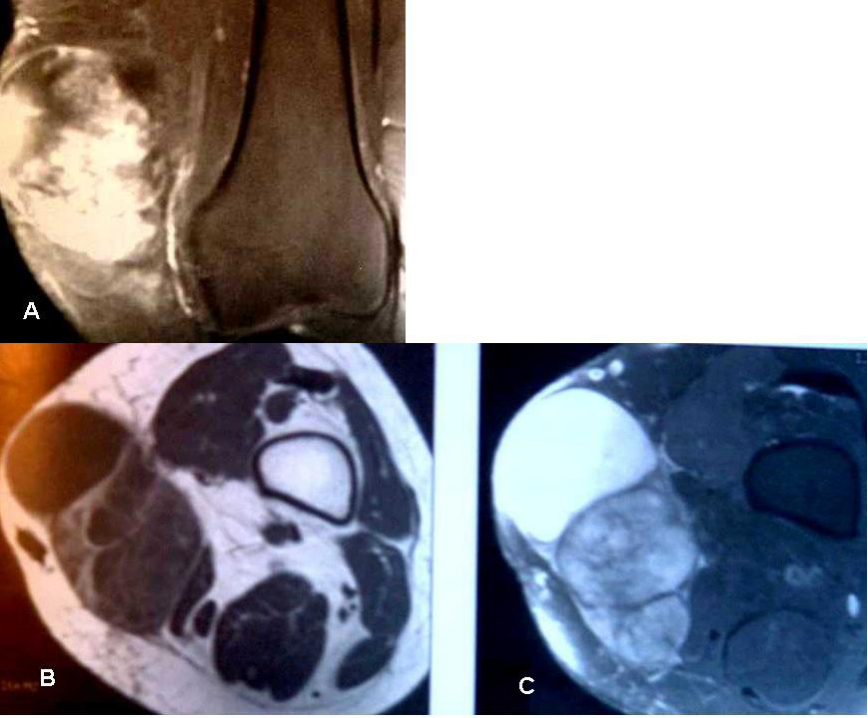
**MRI: 3A, 3B: MRI-Scan of myxoid liposarcoma of the thigh, coronal (3A) and axial (3B) views**.

It is often well defined with little adherence to the adjacent structures. The clinical diagnosis of malignancy of this adipose tumor is thus difficult but findings of an important size (> 5 cms) and rapidly growing mass should alert and lead to the realization of an appropriate preoperative X-Ray investigation (U/S-Scan then MRI-Scan and biopsy) [1,2,14]. The U/S-Scan helps determine the size, the shape and the outlines of the expansive tissular process as well as its ultrasound structure and its homogeneity (fig. 2); it also determines its relationship with the surrounding structures. Its deep location (subaponeurotic) and\or the presence of a central necrosis are bad prognosis factors requiring the need for a MRI Scan which still remains the imaging modality of choice to best define an adipose tumor of soft tissues, delineate its anatomical location and carry out a proper pre-biopsy staging and a well-planned surgical procedure (fig. 3A, B) [1,14]. The LPS appears in spontaneous highsign in level-headedness T1 which disappears in technique of fat suppression (spectral saturation or "Fat Sat"); Myxoid liposarcoma is suspected in the presence of cystic zones [1]. The tumour can be badly limited, even infiltrative however a benign intramuscular lipoma might be very infiltrative and a well-circumscribed tumor does not eliminate a LPS [1,5]. Actually, a fat tumor should be considered as a liposarcoma until proved otherwise when it features septums of more than 2 mm thick and nodules or not fatty areas [14]. In case of suspected soft-tissue tumor, an accurate histological diagnosis should be performed prior to any surgical treatment which allows early carcinolytic excision in case of malignancy. According to the standard surgical procedure, a wide excision should be performed each time the adjacent structures allow it (neurovascular axis). Should the surrounding structures be unfavorable, neoadjuvant chemotherapy and\or radiotherapy could be considered to reduce the tumor size [2,11,15]. A wide excision is performed without seeing the tumor, with a safety margin of at least 2 cm previously planned on the MRI Scan, combined with drainage in the axis of the surgical approach [2].

The operative specimen must be located and oriented, to accurately define the margins of the excision. It is sent to the pathologist, in a fresh state, along with a thorough clinical information mandatory for proper histological analysis (age, extent, location and depth of the tumor, date of appearance, and previous treatment); the type and objective of the surgical procedure are detailed [7]. The myxoid LPS has a well-defined macroscopic aspect with no signs of malignancy. The histological examination reveals a myxoid tumor, of misleading benign appearance, since it is free from cyto-nuclear atypias and demonstrates a very low mitotic activity; the diagnosis is based on the particular aspect of vascularization on the one hand, and on the thorough detection of lipoblasts, on the other hand, typically encountered in malignant tumors of adipose origin [5]. Diagnosis of subaponeurotic tumors of the thigh are easily performed in adults over 40 years old; Nevertheless, it is advisable to eliminate benign tumors of myxoid aspect and more specifically intramuscular myxomas and, in a post-traumatic context, lipomas presenting myxoid degenerative changes or reactional tumor-like lesions such as proliferating myositis [3,5,6]. The presence of areas of round cell dense proliferation might result in an uneasy differential diagnosis with other malignant tumors (melanoma, carcinoma, lymphoma); the diagnosis is dependent upon accurate detection of lipoblasts, possibly helped by immunohistochemical markers (S100 protein) [3,5]. There is a great variety of histopronostic scoring systems; the National Union of Cancer Centers grading system (NUCCGS) is widely used in Europe; it was first described by Trojani and al.[9] then was revised by Guillou and Coindre [8]. This rating system is based on three histological criteria: differentiation, number of mitoses and presence of a tumor necrosis. The prognostic value of cytogenetic analysis determines the risk for local recurrence which is first conditioned by the quality of the surgical excision and, in a lesser measure, the histological grading of the lesion. The analysis of the excision margins is critical; excision margins that exceed one centimeter might be considered as negative; margins under this value are considered as " suspicious " or positive (intra-tumors) [12]. The risk of metastasis and thus the global survival rate mostly depend on the histological pattern [8,10]. In the present case (histopronostic grade 2 featuring more than 10% of round cells), the presence of a great number of round cells is considered as the major predicting factor for prognosis, and prevails over the histological grade, as well as the presence of positive or negative excision margins. Myxoid LPS have a high risk of local recurrence (50%) whereas pure myxoid LPS report a 20% rate of metastasis. At the other extremity of the spectrum of disease, the LPS featuring a majority of round cells, metastasizes in 70% of the cases. These metastases arise in lungs and bone but, also, in serous membranes (pleura, pericardium and peritoneum) [10]. The average survival rate is 80% at 5 years and 50% at 10 years, strongly determined by the quality of the local excision [7,10]. The local reference treatment for soft-tissue sarcomas of the extremities includes the combination of surgery and radiation therapy. The indication should be discussed by a committee of multidisciplinary oncologists; Surgery is usually completed by an adjuvant radiation therapy and, sometimes, by a complementary chemotherapy according to the results of the pathology examination and the general extension of tumor [2,11]. Once the course of treatment has been completed, a necessary schedule of follow-up begins. Clinical examinations, imaging (MRI Scan and TAP-Scan) are performed every six months for five years after treatment, then annually for at least 5 years after this. Since sarcomas are exceedingly rare tumors, our review of the world literature provided very few studies on liposarcomas and even less on myxoid LPS [3,7,10]; The published series include all types of liposarcoma and soft-tissue sarcomas, with a therapeutic protocol based on the histological grade and potential metastases [2,7,10,15]. Despite the reassuring clinical aspect of his tumor, our patient should have benefited from an X-ray investigation and a preoperative biopsy. Moreover, his tumor was voluminous, deep and intramuscular. The histopathologic examination of the operative specimen confirmed the malignancy with positive margins of excision. Dujardin [2] confirms that the isolated excision of a soft-tissue sarcoma exposes the patient to a risk of local recurrence from 50 to 93% according to the type of tumour. The prognosis for this patient is thus reserved since it presented with a myxoid LPS which survival rate is 60% at 5 years [10] which, furthermore, was initially handled by a margin excision. Taking into account the tumor size (7.5 cm), the histological features (number of round cells = 30%), the initial margin excision and the young age of the patient (50 years), re-excision followed by adjuvant radiation therapy and implementation of a complementary chemotherapy was recommended by the oncology committee in order to decrease the risk of local recurrence and metastasis [2,7,10]. Staging studies comprising MRI Scan of the thigh and an abdo-thoracic TAP-Scan, followed by a tumor biopsy should have been undertaken for accurate histological diagnosis. These elements would have helped the oncology committee to suggest a wide surgical excision of the voluminous tumor completed by adjuvant radiotherapy [9].

## Conclusion

The present case clearly illustrates the problems associated with the management of these rare tumors and which might be encountered by any surgeon, whatever the surgical field. Any soft-tissue tumor diagnosed in the adult, particularly when its size exceeds five centimeters and/or when of subaponeurotic origin, typically requires the need for a careful preoperative X-ray investigation combined with a diagnostic biopsy. In case of malignancy, an early accurate medico-surgical treatment should be implemented, associated with a systematic histopathological examination. The prognosis for patients with soft tissue sarcoma largely depends on the quality and coherence of the initial management protocol based on a close multidisciplinary cooperation within the oncology committee.

## Competing interests

The authors declare that they have no competing interests.

## Authors' contributions

FL prepared the draft manuscript. CB helped in preparation of the draft manuscript. FC contributed pathological part of the manuscript and the photomicrographs. All authors read and approved the final manuscript.

## Consent

Patients consent was obtained for the publication of this case report.
